# Diseases of Women, Including Their Pathology, Causation, Symptoms, Diagnosis and Treatment

**Published:** 1882-03

**Authors:** 


**Diseases of Women, including their Pathology, Causation, Symptoms, Diagnosis and Treatment.** A manual for students and practitioners. By ARTHUR W. EDIS, M. D, London, F. R C. P., M. R. C. S., Assistant Obstetric Physician to the Middlesex Hospital, &c. With one hundred and forty-eight illustrations. Philadelphia: Henry C Lea's Sons & Co. 1882.

This new work on the diseases peculiar to women aims to be a reliable, practical and clinical guide to the student and young practitioner in this department. Its publication following so soon after the exhaustive treatises of Thomas and Emmet and Byford, affords the opportunity for comparison which, we are forced to say, is not at all favorable to our English author. The present work is, however, an excellent manual, and presents the various subjects of which it treats in a clear manner. It is evident, however, that in the department of gynecology the American profession are far in advance of our brethren across the water in their comprehension of this special class of diseases and in their skill in devising and applying measures for their relief and cure. The present work is not as complete as Hewitt's, while it possesses the advantage of greater clearness and conciseness in many of its subjects, and therefore better adapted to the student for whom it is written. We commend it, therefore, as a very practical treatise within the reach of most of those who desire a concise work on this subject, and whose tastes do not permit the expenditure of special attention to this or any other specialty.

